# Decoupled learning for brain image registration

**DOI:** 10.3389/fnins.2023.1246769

**Published:** 2023-08-25

**Authors:** Jinwu Fang, Na Lv, Jia Li, Hao Zhang, Jiayuan Wen, Wan Yang, Jingfei Wu, Zhijie Wen

**Affiliations:** ^1^Institute of Infectious Disease and Biosecurity, School of Public Health, Fudan University, Shanghai, China; ^2^China Academy of Information and Communication Technology, Beijing, China; ^3^Industrial Internet Innovation Center (Shanghai) Co., Ltd., Shanghai, China; ^4^School of Health and Social Care, Shanghai Urban Construction Vocational College, Shanghai, China; ^5^Department of Mathematics, School of Science, Shanghai University, Shanghai, China; ^6^College of Intelligence and Computing, Tianjin University, Tianjin, China; ^7^School of Economics, Shanghai University, Shanghai, China

**Keywords:** unsupervised learning, data-adaptive, brain image registration, model decoupling, sub-problems

## Abstract

Image registration is one of the important parts in medical image processing and intelligent analysis. The accuracy of image registration will greatly affect the subsequent image processing and analysis. This paper focuses on the problem of brain image registration based on deep learning, and proposes the unsupervised deep learning methods based on model decoupling and regularization learning. Specifically, we first decompose the highly ill-conditioned inverse problem of brain image registration into two simpler sub-problems, to reduce the model complexity. Further, two light neural networks are constructed to approximate the solution of the two sub-problems and the training strategy of alternating iteration is used to solve the problem. The performance of algorithms utilizing model decoupling is evaluated through experiments conducted on brain MRI images from the LPBA40 dataset. The obtained experimental results demonstrate the superiority of the proposed algorithm over conventional learning methods in the context of brain image registration tasks.

## 1. Introduction

Medical image registration is a vital step in the healthcare field, pivotal for diagnosing (Song et al., 2021), and planning treatments (Tan et al., 2016). It aligns multiple images, establishes spatial correlations, and assimilates varied data, thereby contributing to improved diagnostic precision and personalized treatments.

The task of image registration (Hu et al., 2018), involves identifying the optimal spatial transformation between two images, thereby establishing a unique correspondence between points in each space that are associated with the same anatomical position. This task is a high-dimensional, ill-posed optimization problem, commonly solved using a specific objective function:


(1) $$\begin{array}{l} T^{*}=\mathrm{argmin}D\left(I_{f},T\left(I_{m}\right)\right), \end{array}$$

where T^*^ represents the optimal transformation, *I*_*f*_ is the template (or fixed) image, and *I*_*m*_ is the image to be registered (or moving image). The function *D*(·, ·) quantifies the dissimilarity or distance between these two images.

Traditionally, medical image registration has been conducted with model-based methods. These models are typically categorized into parametric methods and global variational methods. Parametric methods approximate deformations using parameters, such as Thin-Plate Splines (TPS) (Bookstein, 1989) or B-splines (Xia and Liu, 2004), and solve an optimization problem to find optimal parameter values. Conversely, global variational methods frame the registration problem as an energy functional minimization task, often involving partial differential equations to ensure the diffeomorphism of the deformation field. Although these model-based methods offer high registration accuracy and robustness, they suffer from computational complexity and limitations in capturing complex deformations.

Recently, the rapid advancements in deep learning and the availability of extensive medical image datasets have catalyzed the emergence of learning-based registration methods. The early deep learning-based image registration models primarily utilized supervised learning methods. In this approach, output labels such as deformation vector fields or parameters are used during training to learn the mapping from input image pairs to deformation fields using neural networks. Various methods, including convolutional neural network (CNN) and fully convolutional network (FCN) (Sheikhjafari et al., 2022) architectures, have been explored to tackle single-modal or multi-modal registration tasks, rigid registration, and non-linear deformations. But these methods require a large amount of predefined ground truth deformation field labels, resulting in significant manpower costs. To overcome the limitations of supervised learning, unsupervised learning models for image registration have been developed. Rather than necessitating predefined ground truth deformation field labels, these models place reliance on the assessment of similarity between registered images and template images to guide the network learning process. Unsupervised learning models (Sideri-Lampretsa et al., 2022) have demonstrated competitive performance compared to traditional methods, surpassing them in metrics like Dice score, residual sum of squares, peak signal-to-noise ratio, and structural similarity. Despite their promising results, deep learning-based registration methods face certain challenges, including the presence of local minima during model optimization, which can impede convergence to accurate solutions.

To address these existing challenges, this paper bridges traditional model-based methods and modern learning-based deep learning methods, aiming to balance global smoothness and local data-adaptive discontinuity constraints. This combination is anticipated to enhance the accuracy and precision of brain image registration. Specifically, this paper introduces an unsupervised learning method specifically designed for medical image registration, focusing on brain images. The proposed method incorporates a regularization term to tackle the inherent complexity of the registration problem, thus splitting it into more manageable sub-problems through model decoupling techniques. These sub-problems are then addressed via deep learning networks, namely Similarity-Net and Denoiser-Net. Our main contributions include (a) the development of an innovative deep learning method: This novel method uses model decoupling to simplify the inverse problem of image registration. It accomplishes this by decomposing the problem into two less complex subproblems, (b) introduction of a deep learning algorithm based on model decoupling: This proposed algorithm addresses the highly ill-posed problem of image registration. The innovative aspect of this algorithm lies in its ability to utilize deep learning techniques to approximate the solutions to these lower complexity subproblems, and (c) The obtained experimental results demonstrate the superiority of the proposed algorithms over conventional learning methods in the context of image registration tasks.

## 2. Related works

### 2.1. Deep learning based registration methods

Supervised learning techniques in image registration utilize known deformation vector fields during training, with loss functions commonly comprising similarity and regularization terms. The creation of deformation labels can be quite challenging, prompting the use of random generation (Sun et al., 2018), or model-based generation approaches (Yang et al., 2016). While these techniques are valuable, they may encounter limitations due to the general lack of labeled data.

Unsupervised learning approaches (Liu et al., 2022), such as the VoxelMorph network (Balakrishnan et al., 2018), tackle the challenge of obtaining ground truth deformation fields by capitalizing on the similarity between registered images and template images. The VoxelMorph network incorporates the U-Net architecture (Ronneberger et al., 2015) for predicting the deformation field and the Spatial Transform Network (STN) module (Jaderberg et al., 2015) to apply the predicted deformation to the target image. This structure circumvents the need for explicit deformation labels, demonstrating the power of unsupervised learning in accurate image registration.

### 2.2. Regularization based methods

Diffeomorphic regularization, a widely adopted method, preserves the topological structure of images during registration (Beg et al., 2005). Approaches based on stationary velocity fields and architecture-based designs are common in this respect (Trouvé and Younes, 2005; Vercauteren et al., 2009). Recent advancements aim to predict diffeomorphic deformation fields within deep learning frameworks, with some methods, like SYMNet (Lu et al., 2019), directly outputting pairs of diffeomorphic deformation fields. These techniques aim to boost the smoothness and realism of deformation fields, thereby improving the accuracy and efficiency of registration.

Multi-scale regularization techniques, on the other hand, utilize information from multiple scales to enhance the robustness and accuracy of the process. Approaches such as multi-scale information fusion (Srivastava et al., 2022), multi-stage registration (de Vos et al., 2019; Cai et al., 2022), and coarse-to-fine registration (Zhao et al., 2020; Mok and Chung, 2022) have been developed to implement multi-scale regularization. Despite an increased demand for computational resources, these multi-scale techniques have demonstrated superior performance in various medical image registration tasks.

## 3. Method

### 3.1. Model framework

In the context of brain magnetic resonance image registration, it is desired to maintain the topological structure of the images before and after registration. To achieve this, we consider the following optimization problem:


(2) $$\phi^{*}=\arg\;\min\;L_{\text{sim}}(\left(I_{f},I_{m}◦\phi \right)+\text{λ}\cdot \|\nabla \phi {\|}_{2}^{2},$$

where, *I*_*f*_ represents the template image, *I*_*m*_ represents the image to be registered, ϕ denotes the predicted deformation field, and |∇ϕ|^2^ is the regularization term that imposes a smoothness constraint on the deformation field. The parameter λ balances the relationship between the fidelity term and the regularization term in the loss function.

Considering the complexity of image registration problems, the above optimization problem is a high-dimensional and ill-posed problem. Therefore, we propose an optimization method based on model decoupling. By introducing relaxation variables, the above optimization problem is transformed into two sub-problems:


(3) $$u^{*}=\arg\;\min\;L_{\text{sim}}\left(\left(I_{f},I_{m}◦u\right)+\alpha \cdot {\left|u-v\right|}_{2}^{2}\right),$$

(4) $$v^{*}=\arg\;\min|v-u{|}^{2}+\beta \cdot |\nabla v{|}_{2}^{2},$$

where, *v* is the relaxation variable, both *u* and *v* represents the deformation field in this problem and α and β are balancing parameters.

**Figure 1:**
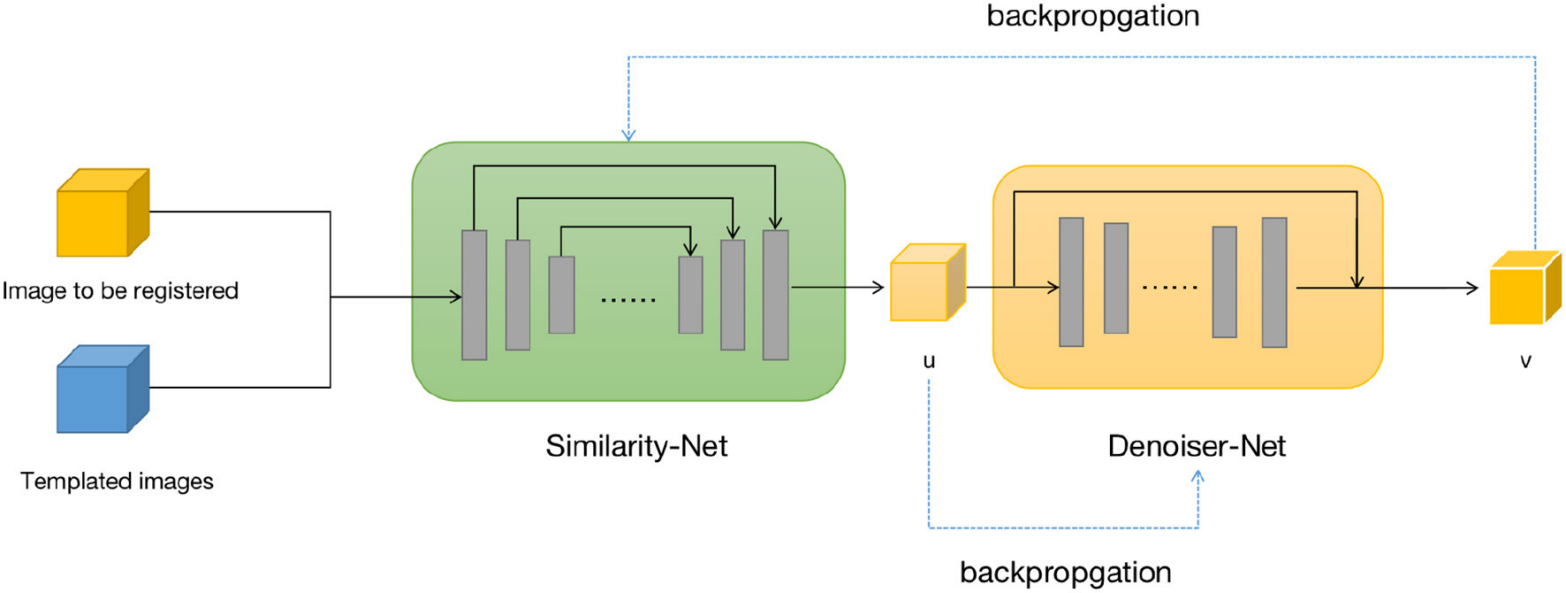
Brain image registration network structure, both *u* and *v* represents the deformation field.

We design two neural networks to solve these two sub-problems. The first sub-problem is primarily addressed by using the Similarity-Net as the registration network, while for the nature of the second sub-problem, we design a denoising network, Denoiser-Net, to approximate the solution. By iteratively alternating between these two networks, a deformation field with smoothness properties is predicted. The model framework is illustrated in Figure 1. Detailed information will be discussed in Sections 3.2 and 3.3.

We provide the specific steps of the model decoupling-based method for solving the registration problem.

**Table:** 

Model-decoupling-based brain image registration method.
Require: Image pairs $\left(I_{f}^{n},I_{m}^{n}\right)$, parameters α, β > 0, iterations *k*, learning rate *lr*, batch size *B*
Ensure: Optimal solutions u^*^, v^*^.
1: Input: $\left(I_{f}^{n},I_{m}^{n}\right),n=1,\cdots \;,N$.
2: Initialization: Network parameters of Similarity-Net and Denoiser-Net, at this point *i* = 0.
3: for *i* ≤ *k* do
4: Randomly select a batch of data $\left(I_{f}^{j},I_{m}^{j}\right),j=1,\cdots \;,B$.
5: Fix the network parameters of the Denoiser-Net, calculate *u* and *v*.
6: Compute loss (3), update Similarity-Net via backpropagation.
7: Fix the network parameters of the Similarity-Net, calculate *u* and *v*.
8: Compute loss (4), update Denoiser-Net via backpropagation.
9: *i* = *i*+1.
10: end for
11: Output: u^*^ = u, v^*^ = *v*.

### 3.2. Similarity-Net

For the first sub-problem, we employ a similar optimization method as VoxelMorph, using a network called Similarity-Net. We adopt a network architecture similar to UNet, but with reduced network parameters and model complexity. In the encoding part, instead of performing a convolution operation with a stride of 1 after downsampling the image size, we introduce a convolution operation with a stride of 2. Additionally, the number of channels in the feature maps is reduced. In the decoding part, we restore the image size gradually using direct interpolation instead of using transposed convolution, aiming to reduce network parameters. In the encoding part of the network, we perform four convolution operations with a stride of 2 and save the corresponding feature maps. In the decoding part, we restore the image size using nearest-neighbor interpolation, and before each interpolation step, we connect the feature maps saved in the encoding part at the corresponding scale. Finally, after two convolution operations, the predicted deformation field is obtained.

**Figure 2:**
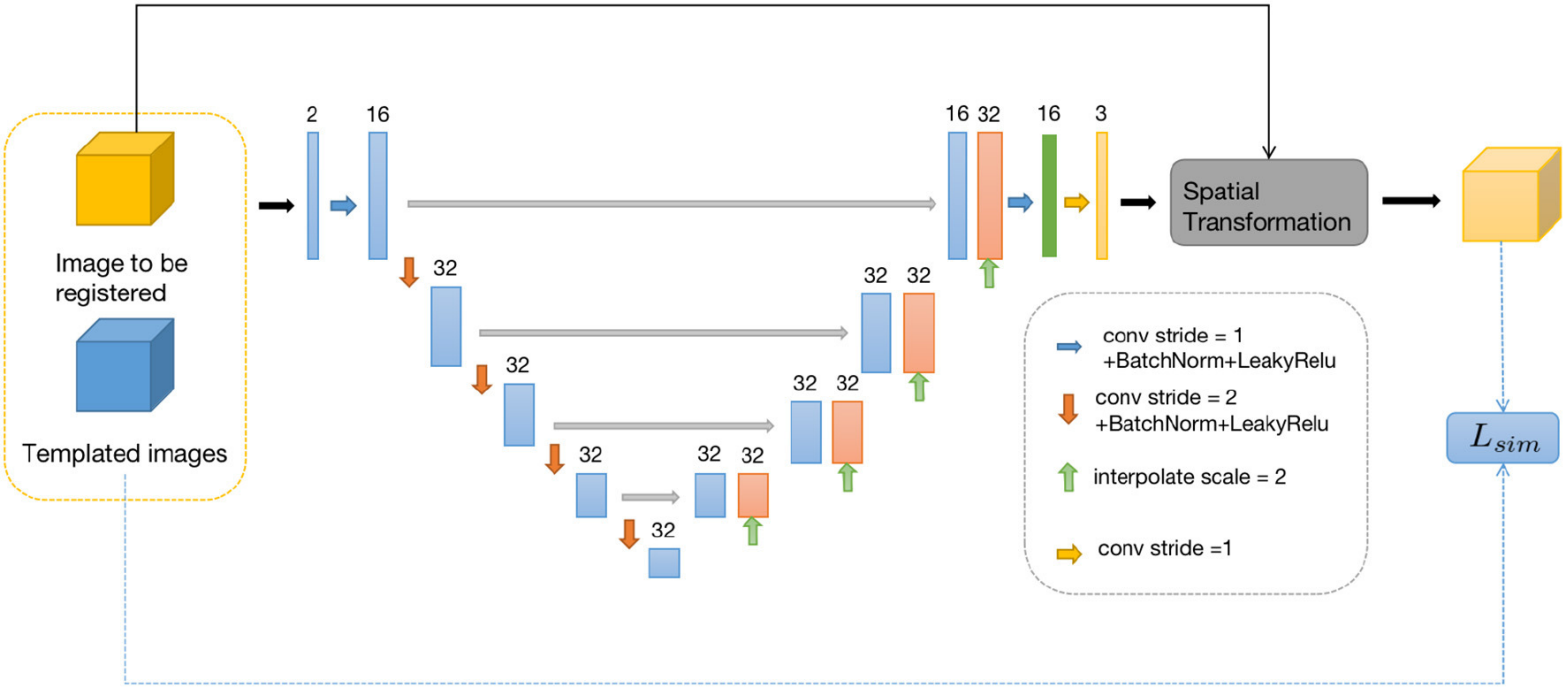
Similarity-Net network framework.

Once the predicted deformation field is obtained, we not only use the spatial transformation layer to register the moving image but also evaluate the distance between the deformed moving image and the template image using local cross-correlation. The deformation field is then fed into the Denoiser-Net network to adjust the deformation field to satisfy the corresponding regularization constraints. The difference between the input and output of the Denoiser-Net is computed as the loss function, which guides the parameter updates of the Similarity-Net. The specific network structure is shown in Figure 2.

### 3.3. Denoiser-Net

The second sub-problem aims to obtain an output that is similar to the input but possesses certain desired properties. This is a common task in image denoising. To address this, we design a small denoising network called Denoiser-Net to solve the second sub-problem. Inspired by DnCNN (Huang et al., 2021) and ResNet (Zhang et al., 2017), we adopt a residual learning approach, where instead of directly mapping the input to the output, we learn the residual between the output and the input. In this design, the relationship between *u* and *v* can be expressed as:


(5) $$\begin{array}{l} v=u+\text{Residual}\left(u\right). \end{array}$$

**Figure 3:**
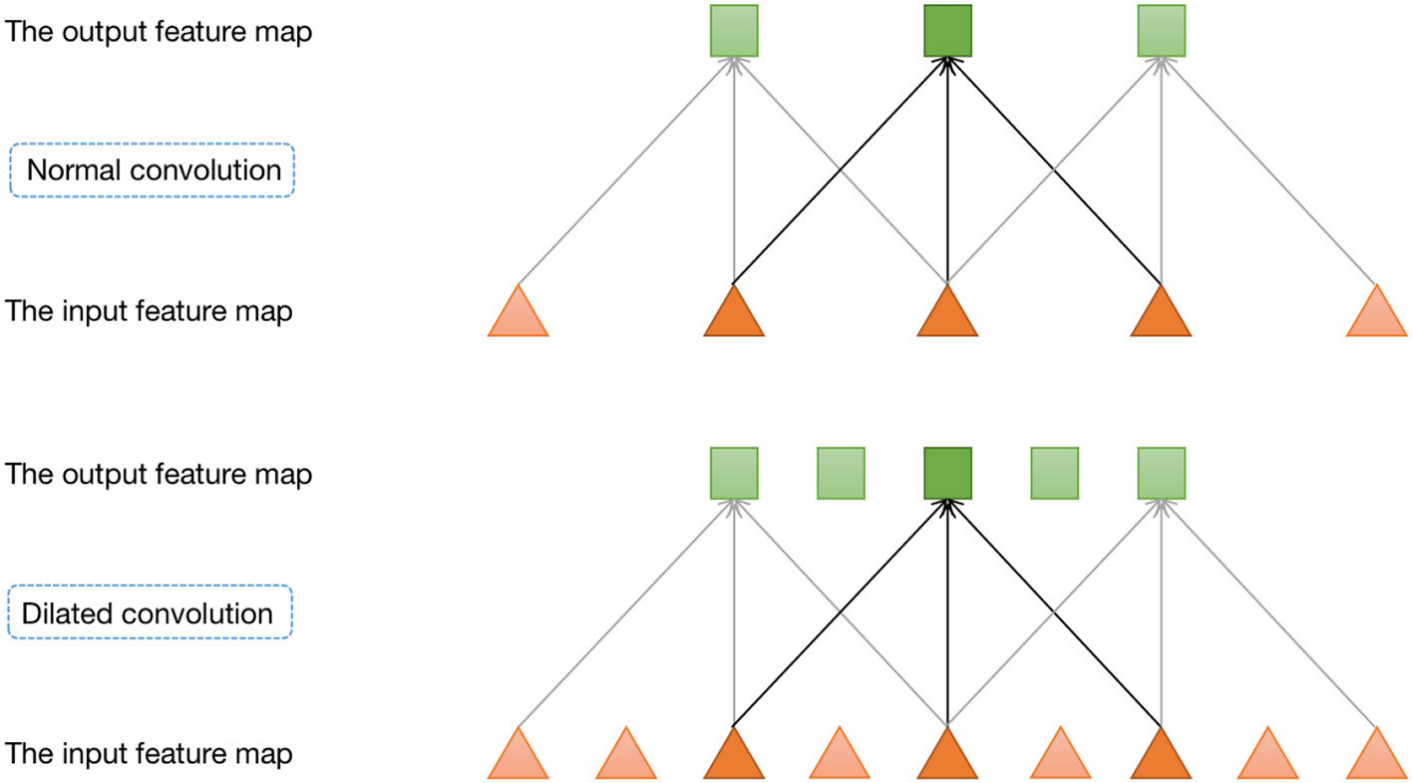
One-dimensional dilated convolution operation.

**Figure 4:**
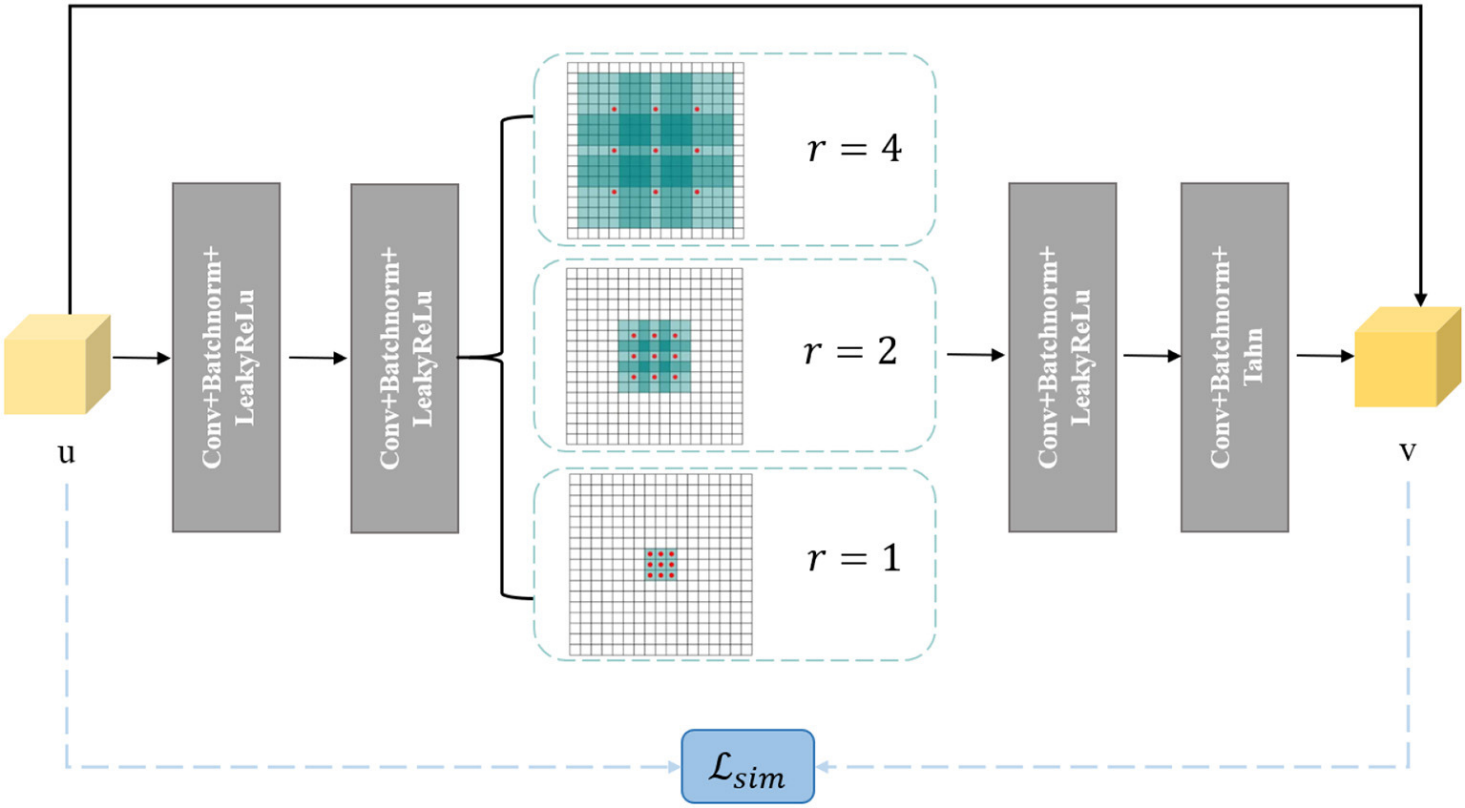
Denoiser-Net network framework.

Furthermore, we incorporate a pyramid structure inspired by SPPNet (He et al., 2016) into the network construction, utilizing parallel dilated convolution operations with multiple dilation rates to achieve multi-scale information fusion. Dilated convolution, also known as atrous convolution, enables explicit control over the resolution of the computed feature maps in convolutional neural networks and allows adjustment of the filter's receptive field to capture multi-scale feature information. It is a generalization of conventional convolution operations. In the case of 1D signals, the dilated convolution applied to the input feature map *x* with the output feature map *y* and convolution filter *w* can be expressed as:


(6) $$\begin{array}{l} y\left[i\right]=\underset{k}{\sum }x\left[i+r\cdot k\right]w\left[k\right], \end{array}$$

where *y*[*i*] represents the value at the *i*-th coordinate position of the output feature map *y*, *r* denotes the dilation rate, and *k* represents the *k*-th position of the filter. Figure 3 provides a visualization of dilated convolution in 1D signals. In conclusion, the specific structure of Denoiser-Net is illustrated in Figure 4.

## 4. Experiments

### 4.1. Data preparation

**Figure 5:**
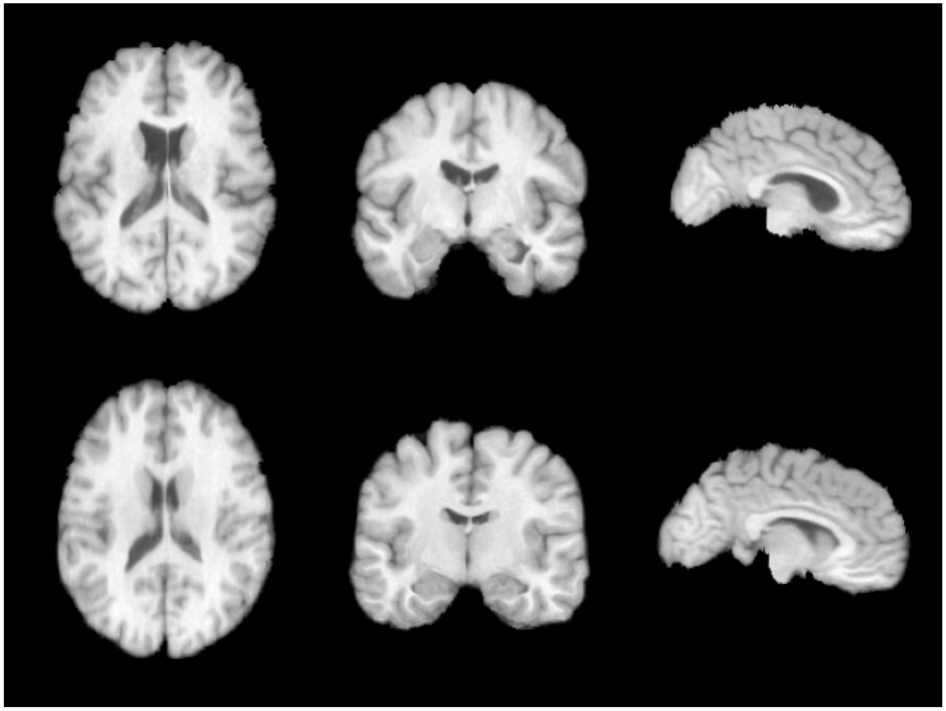
LPBA40 dataset.

The brain image dataset used in this study is the publicly available LPBA40 dataset. The LPBA40 dataset was collected at the North Shore Long Island Jewish Health System (NSLIJHS) and is maintained at the University of California, Los Angeles (UCLA). The dataset consists of 40 brain magnetic resonance imaging (MRI) scans from volunteers, with voxel sizes of 0.86 × 0.86 × 1.5mm^3^. The volunteers include 20 males and 20 females, all free of any brain disorders, psychiatric history, or intellectual developmental delay. The average age of the volunteers is 29.20 ± 6.30 years, with the youngest volunteer being 19.3 years old and the oldest being 39.5 years old. The UCLA Laboratory of Neuro Imaging (LONI) manually labeled 56 brain regions for each image in the LPBA40 dataset. The specific definitions of the brain regions can be found in Zhang and Ghanem (2018). We performed a series of standardization processes on the brain MRI images. Firstly, we used the FreeSurfer software (Shattuck et al., 2008) for skull stripping and resampled the images to a voxel size of 1 × 1 × 1mm^3^. To avoid computational redundancy caused by blank regions in the images, we cropped the images to a size of 144 × 192 × 160mm^3^. To eliminate the impact of grayscale value magnitude and distribution on the experiments, we normalized and histogram-equalized the cropped images. Finally, we applied affine alignment to all the images to ensure the center of study in the non-linear transformations across the brain images. Illustrations of the preprocessed images in three directions on the same slice are shown in Figure 5.

### 4.2. Experimental setup

The experiments were conducted on a Linux operating system, specifically Ubuntu 18.04. The network was built using the PyTorch deep learning framework. The training and testing were performed on an NVIDIA GeForce RTX 3090 GPU with 24GB of memory. To demonstrate the effectiveness of our proposed model-decoupled method on brain data, we compared it with the following methods:

(1) Similarity-Net: The network architecture is Similarity-Net without the regularization term in the loss function and without the inclusion of the denoiser network, which serves as our baseline method. For convenience, we refer to this method as S-Net.
(2) Similarity-Net with Smoothness Regularization (SS-Net): The network architecture is Similarity-Net, and the loss function includes smoothness regularization constraints but does not include the denoiser network.
(3) VoxelMorph: The network architecture is U-Net, which has more parameters than Similarity-Net, and the loss function includes smoothness regularization constraints.

### 4.3. Evaluation metrics

In this study, we used the Dice similarity coefficient (DSC) as a commonly used evaluation metric for quantitatively analyzing the registration performance in brain image registration. The DSC is defined as follows:


(7) $$\begin{array}{l} \text{DSC}\left(A,B\right)=2\frac{\left|A\cap B\right|}{\left|A|+|B\right|}, \end{array}$$

where DSC(*A, B*) represents the degree of overlap between two corresponding brain regions A and B, where A and B denote the brain regions of the template image and the registered image, respectively. The DSC value ranges from 0 to 1, with a higher value indicating a higher degree of overlap and similarity between the two brain structures.

### 4.4. Experimental results

For the experiment, 30 randomly selected images were used as the training set, 2 images as the validation set, and 8 images as the test set for inter-subject brain image registration. This resulted in a total of 870 image pairs available for training. The network was trained with a learning rate of 0.0005, 50,000 iterations, and a batch size of 1.

Table 1 records the Dice Similarity Coefficient (DSC) obtained under different methods. Here, “Ours+” refers to our proposed method, where we replaced the sub-network in the first step with VoxelMorph instead of Similarity-Net and performed alternating iterations with Denoiser-Net. Observing the table, we can draw the following two conclusions: (1) Compared to the method SNet, which only uses Similarity-Net, our proposed method shows a significant improvement in the DSC metric. This indicates that our proposed method effectively imposes regularization constraints on the deformation field, thereby enhancing the registration accuracy. (2) Compared to the method SS-Net, which directly incorporates regularization terms into the loss function, our proposed method also exhibits a slight improvement in the DSC metric. Furthermore, even after replacing Similarity-Net with VoxelMorph, our proposed method still outperforms VoxelMorph, suggesting that our model-based method can further narrow the solution space and reduce the occurrence of local minima to a certain extent.

**Table 1:** DSC of different methods.

**Method**	**S-Net**	**SS-Net**	**Ours**	**VoxelMorph**	**Ours^+^**
DSC	0.6780	0.7027	0.7043	0.7053	0.7061

**Figure 6:**
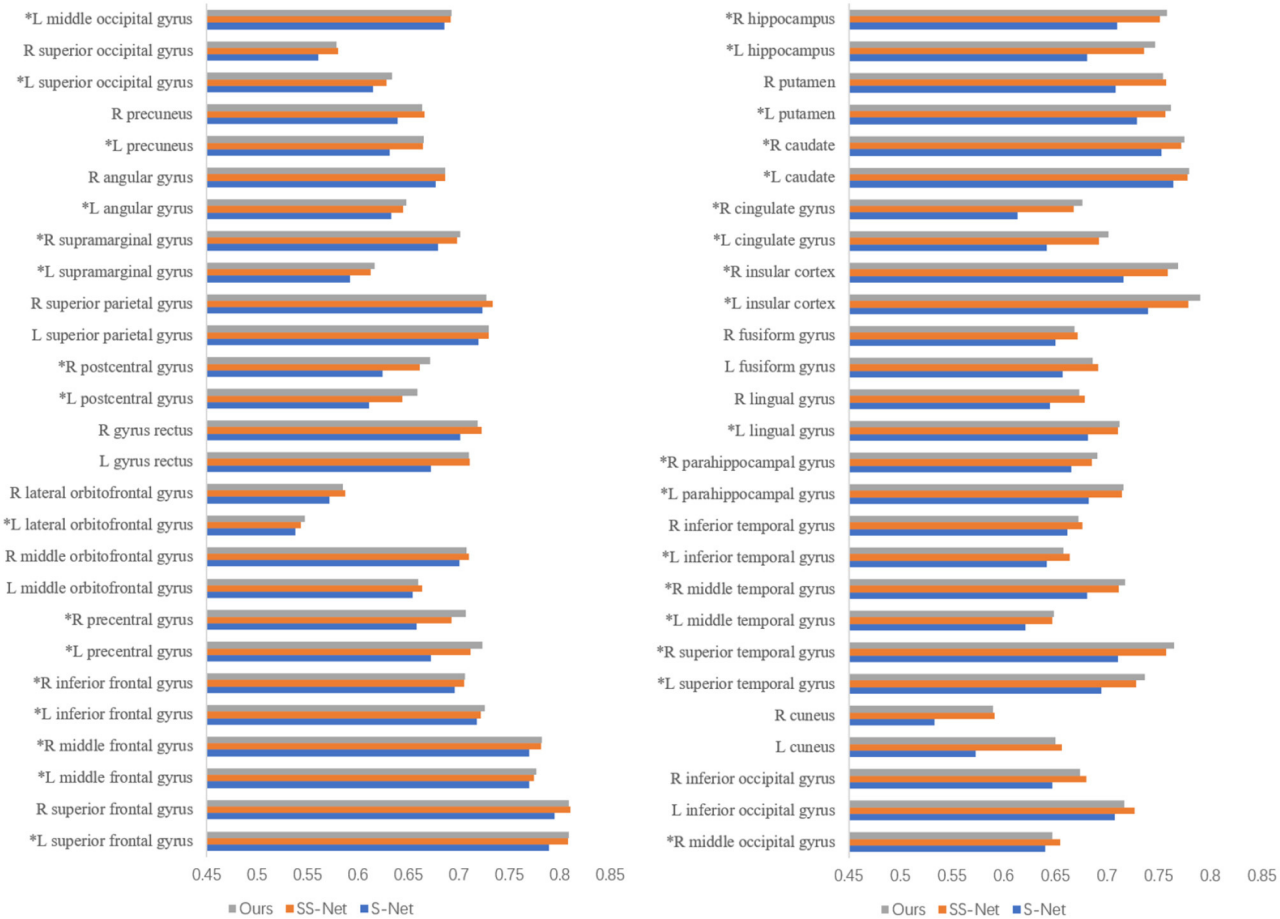
The DSC for different methods in the regions of interest.

Figure 6 presents the DSC (Dice Similarity Coefficient) metrics for S-Net, SS-Net, and our proposed method across 54 regions of interest (ROIs) of interest. The parts marked with asterisks (*) indicate that our method achieved higher DSC values in those brain regions compared to the other two methods. Upon statistical analysis, our proposed method demonstrated superior registration performance in 33 brain regions. This suggests that the improvement in the DSC metric achieved by our method is not limited to specific brain regions but rather reflects an overall enhancement in registration accuracy.

**Figure 7:**
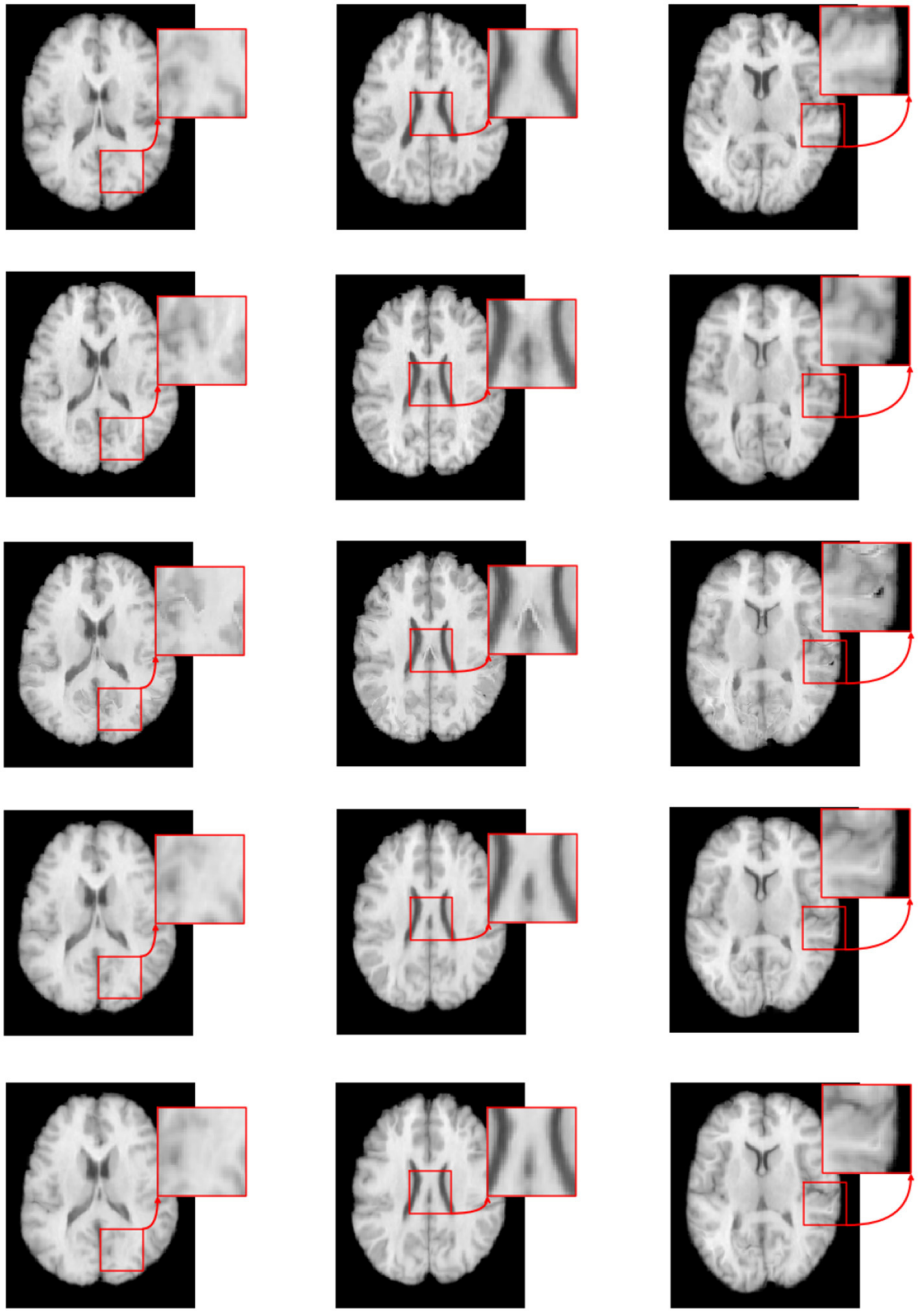
The registered images obtained through different methods.

Figure 7 illustrates the visual results of S-Net, SS-Net, and our proposed method on the LPBA40 dataset. The three columns represent the visualization results for three slices. The top row shows the target (moving) image, the middle row displays the template (fixed) image, the third row depicts the image registered using the S-Net method, the fourth row shows the image registered using the SS-Net method, and the fifth row displays the image registered using our proposed method. By observing the results, it is evident that the image registered using the S-Net method exhibits local discontinuities, connections, and holes that are inconsistent with the actual data. On the other hand, the images registered using the SS-Net method and our proposed method appear smoother and closer to the real data.

**Figure 8:**
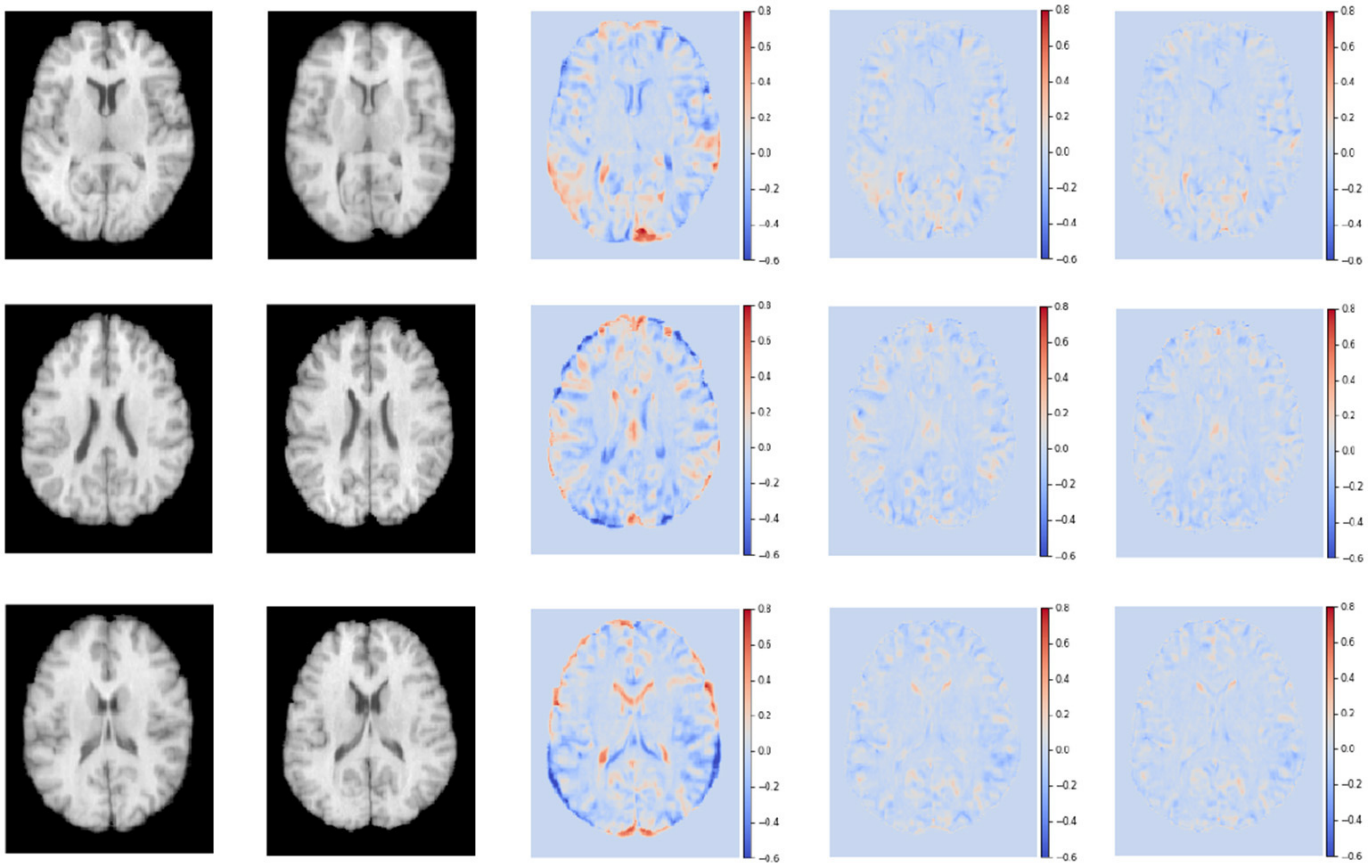
The registered images obtained through different methods.

Figure 8 shows the residual maps of S-Net, SS-Net, and our proposed method on the LPBA40 dataset. The three rows represent the visualization results of three slices. The first column corresponds to the target image, the second column is the template image, the third row shows the difference between the two images without registration, the fourth row shows the difference between the image registered using the SS-Net method and the template image, and the fifth row shows the difference between the image registered using our proposed method and the template image. By observation, our proposed method reduces the differences between the registered floating image and the template image, and in some regions, it performs similarly to or slightly better than SS-Net.

In conclusion, our proposed method outperforms S-Net in terms of evaluation metrics and visual effects, and slightly outperforms SS-Net. This demonstrates that the method based on model decoupling and alternate iterative training strategy effectively learns the smoothness regularization constraint, thereby improving registration accuracy. Furthermore, in the experiments with increased model complexity, i.e., the improved model based on the VoxelMorph framework proposed by us still achieves a certain degree of improvement in performance. This indicates that our method can serve as a framework to be combined with other more sophisticated networks, enhancing registration accuracy on top of the existing network.

## 5. Conclusion

In our study, we propose a novel deep learning method that employs model decoupling to augment the precision of registration tasks in medical imaging. By constructing separate networks for fidelity and regularization terms, we achieve effective constraint of the solution space, thereby reducing the occurrence of local minima that might compromise result quality. Our method's superior performance was demonstrated through its application to image registration tasks on brain magnetic resonance imaging (MRI), enhancing the accuracy of image processing and analysis.

Although our research has made considerable strides in the domain of image registration, there remain potential areas for future exploration. One such aspect pertains to the performance of the two subnetworks within our model. Given the dependency of our unsupervised learning method's registration accuracy on the first network's output, investigating the integration of potentially more efficient network architectures into our framework could be beneficial. This could pave the way for elevated overall registration accuracy.

In terms of regularization, while our work leverages the common differential diffeomorphic regularization for brain MRI datasets, alternative regularization constraints could be explored to further refine the results. This offers another promising avenue for more comprehensive research in the future. By delving into these areas, we anticipate building on our existing contributions and facilitating further advancements in the field of brain image registration through deep learning.

## Data availability statement

The original contributions presented in the study are included in the article/supplementary material, further inquiries can be directed to the corresponding author/s.

## Author contributions

JF spearheaded the project's conceptualization, methodology, and manuscript drafting. NL majorly handled data analysis and interpretation and also assisting in manuscript writing. JL contributed to experimental design and implementation. HZ assisted with data interpretation, manuscript writing, and providing critical feedback. JWe and WY undertook experiments and data acquisition and aided in manuscript revision. JWu and ZW led project management, influenced experimental design, data interpretation, and manuscript writing. All authors approved the final version of the article.
